# Antimicrobial Peptides for Topical Treatment of Osteomyelitis and Implant-Related Infections: Study in the Spongy Bone

**DOI:** 10.3390/ph11010020

**Published:** 2018-02-16

**Authors:** Pavel Melicherčík, Ondřej Nešuta, Václav Čeřovský

**Affiliations:** 1 Department of Orthopaedics, First Faculty of Medicine, Charles University in Prague and University Hospital in Motol, V Úvalu 84, 150 06 Prague 5, Czech Republic; Pavel.Melichercik@fnmotol.cz; 2 Institute of Organic Chemistry and Biochemistry of the Czech Academy of Sciences, Flemingovo nám. 2, 166 10 Prague 6, Czech Republic; nesuta@uochb.cas.cz

**Keywords:** antimicrobial peptides, osteomyelitis, implant-related infections, bone cement, femur heads

## Abstract

We examined the benefits of short linear α-helical antimicrobial peptides (AMPs) invented in our laboratory for treating bone infection and preventing microbial biofilm formation on model implants due to causative microorganisms of osteomyelitis. For this purpose, we introduced a model of induced osteomyelitis that utilizes human femur heads obtained from the hospital after their replacement with artificial prostheses. We found that the focus of the infection set up in the spongy part of this bone treated with AMP-loaded calcium phosphate cement was eradicated much more effectively than was the focus treated with antibiotics such as vancomycin or gentamicin loaded into the same cement. This contradicts the minimum inhibitory concentrations (MIC) values of AMPs and antibiotics against some bacterial strains obtained in standard in vitro assays. The formation of microbial biofilm on implants made from poly(methylmethacrylate)-based bone cement loaded with AMP was evaluated after the implants’ removal from the infected bone sample. AMPs loaded in such model implants prevented microbial adhesion and subsequent formation of bacterial biofilm on their surface. Biofilms did form, on the other hand, on control implants made from the plain cement when these were implanted into the same infected bone sample. These results of the experiments performed in human bone tissue highlight the clinical potential of antimicrobial peptides for use in treating and preventing osteomyelitis caused by resistant pathogens.

## 1. Introduction

Despite improving prevention, operating technique, and postoperative care, as well as broad availability of antibiotics, osteomyelitis and prosthetic joint infections remain among the most serious complications in orthopaedics and traumatology affecting millions of people worldwide each year. Treatment of osteomyelitis is based on local antimicrobial therapy using antibiotics currently available in combination with surgical treatment and adjuvant therapy [1,2,3]. A key problem is treatment of the “dead space” remaining within the bone after the extensive debridement. One of the ways currently used for increasing the therapeutic potential is to fill this cavity, wherein the bacteria are still settled in the form of biofilm [3], with local carriers loaded with antibiotics [2,3,4]. Long-term release of antibiotics from the carriers into the surrounding infected tissue results in high local antibiotic concentration, which greatly exceeds the systemic antibiotic concentration. A number of studies using different types of carriers and different types of antibiotics have been published [2,3,4,5]. At present, the most commonly used local carriers for antibiotics in orthopaedics are calcium sulfate, calcium orthophosphate, collagen, bone grafts, and poly(methyl methacrylate)-based bone cement. Bone cement is currently used as a standard means for the fixation of joint replacements as well as a carrier for antibiotics [6,7,8,9].

The causative microorganisms of osteomyelitis, such as methicillin-resistant *Staphylococcus aureus* (MRSA), *Staphylococcus epidermidis*, *Streptococcus* spp., *Enterococcus faecalis*, and *Pseudomonas aeruginosa*, can often develop antibiotic resistance that complicates healing processes [10]. Particularly alarming is the emergence of *S. aureus* strains exhibiting resistance to vancomycin, which is frequently used as a drug of last resort. These bacteria may also colonize orthopaedic implants and adhere to them in the form of biofilms within which they are protected from the immune system and antimicrobials [11,12,13]. Such periprosthetic infections can lead to implant loosening and arthrodesis.

Because the current antibiotic therapy options may be soon limited for these resistant bacteria [10], the invention of antimicrobials that would not develop resistance of bacteria against them and will be able to bypass the biofilm barrier is one of the ways aimed to reduce the negative impact of orthopaedic infections. Among these, antimicrobial peptides (AMPs) constitute a family of anti-infective agents, which have been studied in recent decades as a promising supplement to, or substitute for, conventional antibiotics [14,15,16]. The overall positive charge and amphipathic secondary structure of their molecules allow AMPs to interact with the negatively charged phospholipid bilayer of bacterial cell membranes, causing its disruption via pore formation or detergent-like disintegration [17,18,19]. Because of this unique mechanism of action, which differs from those of conventional antibiotics, AMPs are less prone to trigger resistance of microbes against them than antibiotics. Moreover, some of them have ability to kill bacterial cells within biofilm [20]. Incorporation of AMPs into local carriers instead of or in combination with antibiotics appears to be a promising approach for combating bone infections [21,22,23,24,25]. AMPs may also be considered for preventing biofilm formation on the surfaces of artificial materials used in orthopaedics [26,27].

In our laboratory, we have invented several novel AMPs that were originally isolated from the venom of various wild bees [28,29]. These peptides of 12 to 19 amino acid residues in length may have potential for use as easily synthesizable agents effective in controlling bone infections and preventing adhesion of microbes to orthopaedic implants, especially when mixed with a carrier for topical drug delivery. 

In this study, we evaluated the efficacy of selected peptide analogues in a model of induced osteomyelitis in which the peptides were released from calcium phosphate cement into the infected spongy human bone sample to eradicate the induced infection. In another experimental setup, we examined the effect of these peptides released from model implants made from poly(methyl methacrylate)-based bone cement on the prevention of bacterial adhesion and consequent biofilm formation on their surfaces while being exposed to microbes inside the infected bone sample.

## 2. Results

### 2.1. Design of Peptide Analogues, Their Biological Activities and Structure

In our investigation of antimicrobial peptides isolated from the venom of wild bees, we identified, among others, two halictines, HAL-1 and HAL-2 [29]. These are dodecapeptides rich in cationic and hydrophobic residues, show potent antimicrobial activity, and are listed in Antimicrobial Peptides Database under APD ID 01922 and 01923, respectively [30]. Halictines are among the shortest AMPs found in nature, which makes them advantageous in terms of their potential for chemical modification and possible applications. Here, we prepared a new series of analogues (Table 1) related to HAL-2 (GKWMSLLKHILK-NH_2_). These analogs, compared to those already studied [29], show lower toxicity to eukaryotic cells while still retaining high antimicrobial activity against most pathogenic microbes that we have tested. We used haemolytic activity determination as a standard testing of AMPs toxicity. We synthesized analogues in which selected amino acid residues in the sequence were replaced by other residues with the aim of modifying their cationicity, hydrophobicity, or helicity (for rationale and interpretation see [29]). Particularly the replacement of Ser5 and His9 in HAL-2 by Lys produced analog I (Table 1) possessing high antimicrobial activity practically against all pathogens tested included *C. albicans* with MIC values ≤ 10 μM (Table 2). Five Lys residues in its sequence make this peptide strongly cationic (net charge +6), which in the combination with six hydrophobic amino acid residues renders its antimicrobial properties. However, its toxicity to human red blood cells determined as LC_50_ = 339 μM in a haemolytic test was considered not to be fully satisfactory (Table 2).

The substitution of Met4 by hydrophobic Leu or Val led in the first case (peptide V) in the sharp increase of haemolytic activity while in the second case (peptide VI) this led to the opposite effect. This was quite surprising since the physicochemical properties of peptides V and VI are very similar. The analog (VI) exhibited comparable antimicrobial activities to peptide I but had lower haemolytic activity. 

Furthermore, we prepared a series of analogues derived from peptide I in which each of the 11 l-amino acid residues in the sequence was successively replaced by its D-enantiomer to follow the effect of the conversion of amino acid configuration on the α-helicity of the peptide and consequently on its biological activity. This d-amino acid residue scan resulted in the analogues, mostly with weak or moderate antimicrobial activities but also with low haemolytic activities (>400 μM) (not shown) clearly reflecting the α-helical structure distortion and its detrimental impact on antimicrobial activity. The analog VIII with the substitution of Leu in the position 7 by d-Leu, which was included in this study, showed, on average, acceptable antimicrobial activity and also good activity against *C. albicans*. The replacement of properly selected l-amino acid residue with its d-enantiomer in this analog plays a key role in the alteration of its helicity resulting in the distinction between bacterial and eukaryotic cell membranes. Shortening of the peptide chain (analog IX) had a favorable effect on its haemolytic activity without substantial effect on its antimicrobial activity. The reason for low antibacterial activity of these analogues against *E. faecalis* is probably due to degradation of peptides by proteases secreted by this bacterium [31].

### 2.2. Efficacy of AMPs in Experimental Model of Bone Infection

**Figure 1:**
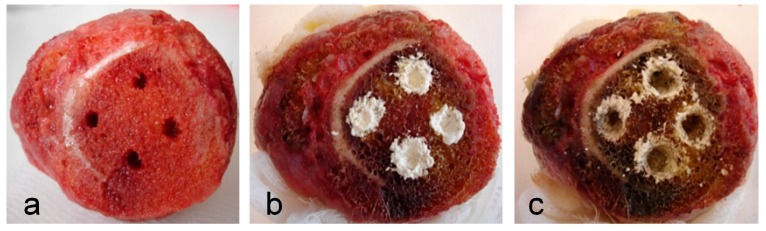
Femur head (**a**) with drilled holes infected with bacteria; (**b**) filled with a peptide blended with local carrier (ChronOS Inject), local carrier alone, and local carrier loaded with antibiotic; (**c**) after removal of the fillings (representative example).

We usually drilled three to four holes into the spongy part of each bone sample (as space allowed) and then filled the holes with the suspension of microbes (Figure 1a). The next day, the foci of the infection set up in the holes were treated (i) with carrier alone as a control, (ii) with carrier blended with AMP, or (iii) in some cases with a carrier blended with an antibiotic for comparison (Figure 1b). The results of infection eradication were evaluated after two days by counting the bacterial colonies (Figures S1–S3 as illustrative examples) found in the swab taken from each hole after the fillings were removed (Figure 1c).

The bone samples as obtained were of different sizes, shapes, compactness, textures, ages, and medical history. Some of the bones evidently had a different consistency, even within the area of the resections that were used for drilling the holes. The appearance of the bones, various strains of bacteria and various peptides and antibiotics to be tested, all together represented a large number of variables. In order to obtain statistically significant data, we decided to select only one peptide (VI), two antibiotics (vancomycin and gentamicin) and two strains of bacteria (MRSA 6271 and *S. epidermidis*) for testing in repeated experiments. For these experiments, we carefully selected femur heads having, as closely resembling as possible, textures of their spongy parts of the bone (Figure 1 as a representative example).

**Figure 2:**
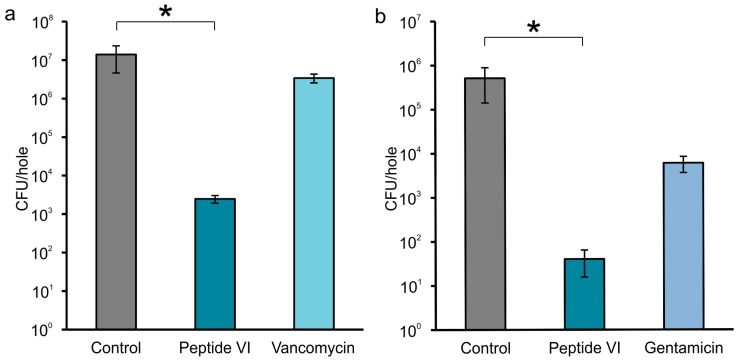
Antimicrobial activity of peptide VI compared to antibiotics against *S. aureus* (MRSA 6271) and *S. epidermidis* (clinical isolates) assessed in the bone samples. The infected holes were treated with the ChronOS Inject carrier (200 mg) blended with peptide VI (10 mg) or antibiotics (10 mg). Carrier alone was used as a control. (**a**) peptide VI vs. vancomycin against *S. aureus*; (**b**) peptide VI vs. gentamicin against *S. epidermidis*. The bars represent mean value ± SEM at least from three independent measurements. Statistical differences between groups were calculated using one-way ANOVA (* *p* < 0.05).

The results of these experiments, as shown in Figure 2a,b, clearly demonstrate that, contrary to the antibiotic (vancomycin or gentamicin), peptide VI used for the treatment of both staphylococcal infections significantly reduces (*p* < 0.05) the bacterial load in the holes when compared to the control.

Due to the different appearances of bone samples and their limited availability, we tested the effect of other peptides I–XII only randomly in single experiments without the possibility of statistical evaluation. The results of the effects of these peptides mixed with the carrier on different bacteria settled in the spongy bone tissue are summarized in Table 3.

Regardless of missing statistical evaluation and possible inhomogeneity in bacterial dissemination, obtained results (Table 3) show that the bacterial counts in the holes treated with the local carrier blended with a peptide were at least three orders of magnitude lower as compared to those in the infected holes that were treated with plain carrier as a control. The treatment of the infection with antibiotics was apparently less efficient as compared to that of AMPs, similarly as illustrated in Figure 2.

### 2.3. AMPs Preventing Biofilm Formation on the Surface of Model Implants

**Figure 3:**
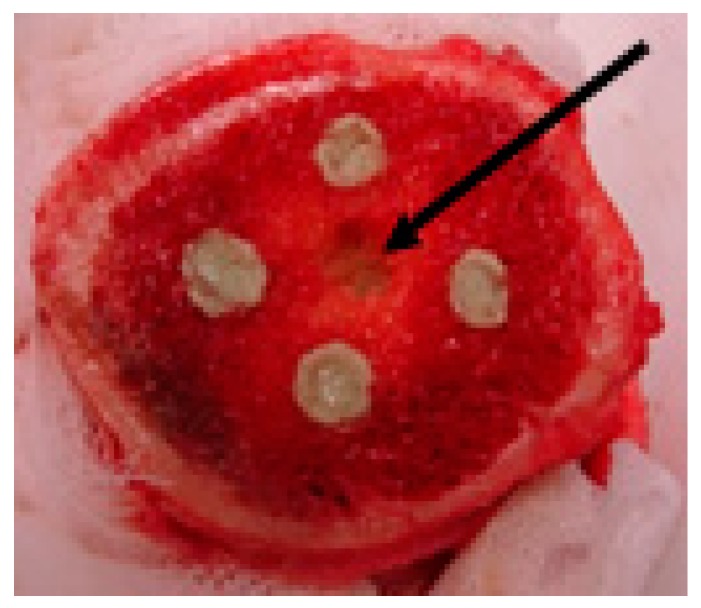
Femur head with inserted implants prepared from poly(methyl methacrylate)-based bone cement (Palacos®r, Wehrheim, Germany) loaded with a peptide and from plain bone cement. Bacteria that were pipetted to the hole between them (arrow) infiltrated through the spongy part of the bone towards the implants, adhered to them and subsequently either formed a biofilm on their surfaces or did not (example representative of the experiments).

The poly(methyl methacrylate)-based bone cement with or without AMPs was implanted either inside infected holes or inside non-infected holes. In the latter case, the microbes infiltrated towards the implant from a hole made between them at roughly equal distance from each (Figure 3). This would simulate the authentic situation in which the pathogens can infect an orthopaedic implant by spreading through the bloodstream or contiguously from areas of infection. We evaluated the experiments on the basis of the amounts of microbes released from the biofilms supposedly formed on the surfaces of the implants that were removed from the infected bone samples. To confirm these results with statistically relevant data, we selected clinical isolate MRSA (M), peptide (VI) and vancomycin for testing in repeated experiments.

**Figure 4:**
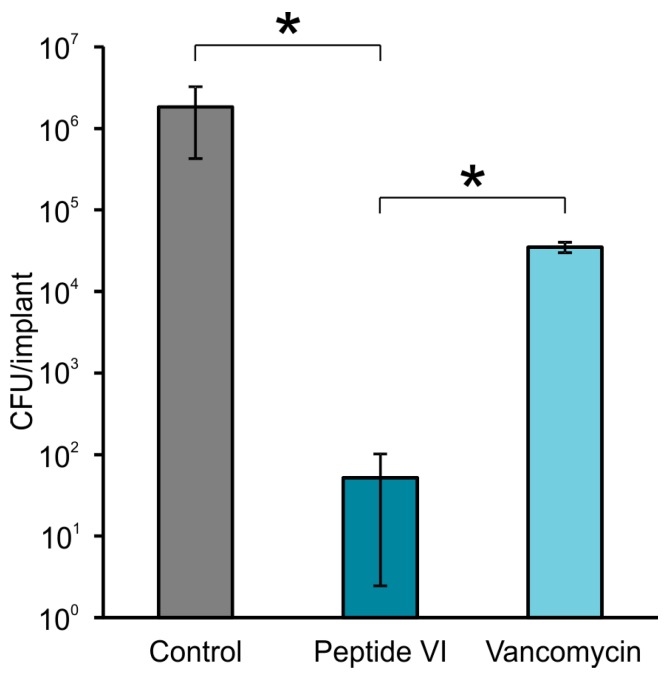
Peptide VI compared to vancomycin in preventing clinical isolate MRSA (M) adhesion and consequent biofilm formation on the implant’s surface made from poly(methyl methacrylate)-based bone cement (Palacos®r). The bone cement contained peptide VI (5 mg), vancomycin (5 mg) or was unloaded as a control. Bacteria infiltrated towards the implants from a hole drilled into bone tissue between them. The bars represent mean value ± SEM at least from three independent measurements. Statistical differences between groups were calculated using one-way ANOVA (* *p* < 0.05).

As shown in Figure 4, the peptide VI loaded to bone cement significantly reduced (*p* < 0.05) biofilm formation on its surface as compared to control as well as to vancomycin loaded cement. The other peptides I–XII incorporated into the bone cement were tested for their ability to prevent biofilm formation on its surface in single experiments, as summarized in Table 4. They evidently reduced adhesion of microbes and consequent formation of a biofilm (see Figure S4 as an example).

The implants prepared from plain cement, on the other hand, always showed high microbial counts related to the biofilms formed on their surfaces. Except for amphotericin B, the effect of antibiotic and antifungal drugs incorporated into bone cement in preventing biofilm formation was lower than the effect of the peptides. The studied peptides also exhibited better effectiveness in preventing the formation of candida biofilms than did the azole antifungal drugs fluconazole and clotrimazole.

## 3. Discussion

In several cases when an all-D isomeric peptide alone, or in combination with its parent L-isomer in a 1:4 ratio in the blend with the carrier were applied to the infected hole, we observed a zero microbial count. We suppose that the contribution of D-isomeric peptides (II, VII, X, XII) to bacterial eradication was due to their resistance to proteases either exerted by bacteria or originated from the cells of the bone tissue. In these cases, we detected intact all-d peptide in the extract of the fillings (recovered from the infected hole after completing the experiment) by Reversed-Phase (RP)-HPLC analysis (not shown). On the other hand, the RP-HPLC analyses of the extracts of the fillings containing solely l-peptides indicated partial to complete digestion of peptides by proteases. Nevertheless, we assume that in all cases presented in Table 3 and Figure 2, it was the fast killing effect of AMPs on bacteria that played a conclusive role in eradicating the infection in the bone samples. When carrying out the experiments, we had to take into account that the bacterial load in an infected hole also could be reduced by components of the bone marrow immune system, including blood cells such as neutrophils. In a few cases, we observed that the bacteria did not grow in holes to which they had been applied the previous day. To find the culprit, we extracted the tissue removed from the holes and fractionated the extract by RP-HPLC (Figure S5a). In one of the RP-HPLC peaks, we detected by mass spectrometry the presence of three human α-defensins HNP1, HNP2, and HNP3 [32] (Figure S5b). These apparently originated from the neutrophils known to be produced in bone marrow and thus may contribute to the eradication of bacteria. In drop diffusion tests [28] (not shown), they exhibited antimicrobial activity against *Micrococcus luteus*, thus indicating the preserved immune system viability in the bone tissue.

We demonstrated here that the peptides exhibit higher efficiency in the eradication of the infection inside the bone sample than vancomycin or gentamicin, despite the fact that the antibiotics exhibited greater activity against planktonic cells (Table 2) when tested in in vitro setup. Vancomycin is known to act on Gram-positive bacteria by inhibiting their cell wall synthesis, but the bacteria are able to develop resistance against it [33].

Gentamicin irreversibly binds to a part of the 30S subunit of the bacterial ribosome, thus interrupting protein synthesis [34]. On the other hand, the killing mechanism of these peptides resides in rapid mechanical breakage of the bacterial cell membrane, independent of bacterial metabolic activity or growth status [17,18,19]. We could speculate that the reason for the higher efficacy of peptides against antibiotics observed in this study might be: (i) the faster killing action of peptides; (ii) in some cases, resistance of microbes against antibiotics; or (iii) different penetration of antibiotics into the surrounding spongy bone tissue as compared to that of the peptides.

Another issue might be the toxicity of these peptides when applied locally to the bone tissue of laboratory animals or humans. As we have already published [35], we did not notice any signs of local or systemic toxicity in laboratory rats when their infected femurs were treated with peptide I. These animals did not have higher degree of swollen limbs or tissue necrosis in comparison to the untreated groups of animals. None of these animals died.

The limitation of this study is that the extent of penetration of peptides and antibiotics to the surrounding bone tissue as well as their release kinetics from the bone cement to the tissue cannot be exactly assessed. Nevertheless, several studies dealing with the release kinetics of different antibiotics and antimicrobial peptides from poly(methyl methacrylate) beads have been published [6,21]. In those experiments, the antimicrobials were released into aqueous media—the setups that neither correspond to the conditions used in our experiments nor the conditions of in vivo reality. Nevertheless, in two parallel experiments, using RP-HPLC methodology, we followed the release kinetics (not shown) of peptide VI and vancomycin from poly(methylmethacrylate) bone cement submerged in physiological solution. The kinetics showed that 17.1% of peptide and 6.1% of vancomycin were released into the physiological solution within 48 h. The slower release of vancomycin into the physiological solution reflects its higher hydrophobicity compared to more hydrophilic peptide, which is more readily released into the aqueous media. This experiment, however, does not reflect the situation in the bone marrow. The femur heads obtained prevalently from older patients contain the so-called yellow marrow, containing up to 50% fat [36]. The release of the peptide or antibiotic from the bone cement to the fatty environment of this bone tissue must be diametrically different to that of the release to aqueous media. In this case, we could anticipate a more favorable release of hydrophobic vancomycin compared to that of peptides.

Furthermore, we need to consider that the conditions in the in vitro tests (determination of MIC values) deviate significantly from the conditions of the experiment performed in the bone. In particular, the concentrations of bacteria in the infected area of the hole may not be comparable to those used in standard in vitro assays. In cases of in vitro antimicrobial assays, the concentration of bacteria is well defined and continuously decreases during the assay due to the action of the antimicrobial agent, while, in our model of osteomyelitis, their concentrations in the area of the infected hole and in the surrounding tissue are unpredictable due to their infiltration into surrounding tissue. We also need to consider that the bacteria adhere to the wall of the hole and supposedly form biofilm on its surface, which makes them more resilient [3]. On the other hand, the total concentration of the peptide inserted in the bone tissue (counted per volume of the hole) exceeds by several orders of magnitude the peptide concentration used for in vitro testing.

## 4. Materials and Methods 

### 4.1. Bone Samples and Microbial Strains

Femur heads (ca. 70 pieces) taken from patients during hip replacement surgery were periodically purchased in the period between 2014–2017 as discarded from the bone tissue bank at University Hospital in Motol, Prague, Czech Republic. The reason for the replacements by artificial prosthesis was the worn out cartilage on the surface of their heads. Bone samples were stored at −80 °C under standard conditions of certified tissue banks. In the laboratory, they were kept at +5 °C overnight and then at room temperature for several hours before they could be used for the experiments. The experiments with this material were approved by the Ethics Committee for Multi-Centric Clinical Trials of the University Hospital Motol, Reference No: EK-655/15. Methicillin-resistant *S. aureus* (MRSA 6271) ATCC 43300 and *P. aeruginosa* (5482) ATCC 27853 were purchased from the Czech National Collection of Type Cultures, National Institute of Public Health, Prague, Czech Republic. Methicillin-resistant *S. aureus* (MRSA (L)) and *P. aeruginosa* (L) were obtained as multi-resistant clinical isolates from Liberec Regional Hospital, Liberec, Czech Republic. Methicillin-resistant *S. aureus* (MRSA (M)), *E. faecalis* and *S. epidermidis* were obtained as clinical isolates from University Hospital in Motol, Prague. *Candida albicans* (F7-39/IDE99) came from the mycological collection of the Faculty of Medicine, Palacky University, Olomouc, Czech Republic. *C. albicans* ATCC MYA-2876, *C. tropicalis* ATCC 750 and *C. glabrata* DSY565 were kindly provided by Dr. Hana Sychrová from the Institute of Physiology, Academy of Sciences of the Czech Republic, Prague. Vancomycin hydrochloride, gentamicin sulfate, ceftriaxone disodium salt, amphotericin B, fluconazole, and clotrimazole were obtained from Sigma-Aldrich, Prague, Czech Republic. 

### 4.2. Peptide Synthesis

Peptides were synthesized manually according to the standard N^α^-Fmoc protocol on a Fmoc-Rink Amide MBHA resin in 5 mL polypropylene syringes having a Teflon filter on the bottom. Crude peptides were then purified by reversed-phase high-performance liquid chromatography (RP-HPLC) as described [28]. The purity of peptides checked by analytical RP-HPLC carried out on an Agilent Technologies 1200 series module (Waldbronn, Germany) with a Supelco C-18, 250 × 4.6 mm column (Bellefonte, PA, USA) ranged from 96% to 99%. The retention times and results of mass spectrometry analyses confirming the identity of peptides are shown in Table 1.

### 4.3. Antimicrobial Activity Determination and Haemolysis Assay

MIC values for peptides were established by observing growth of treated bacteria in HONEYCOMB 100-well microtiter plates (Helsinki, Finland) using a Bioscreen C instrument (Oy Growth Curves, Helsinki, Finland) as previously described [28,29]. Haemolytic activity of peptides expressed as the concentration of a peptide required for lysis of 50% of human erythrocytes in the assay (LC_50_) was measured using a Tecan infinite M200 PRO reader (Tecan Austria GmbH, Grödig, Austria) [28,29]. Blood samples for determining haemolytic activity were obtained from healthy volunteers.

### 4.4. Efficacy of AMPs in Experimental Model of Bone Infection

Three to four holes ca 4 mm in diameter and 8 mm deep were drilled using a surgical spoon at the side of the resection into the spongy part of the femur head. This bone was placed into a beaker cushioned with sterile gauze, which was then drenched with sterile physiological solution. The suspension of selected bacteria (*S. aureus*, *S. epidermidis*, *P. aeruginosa*) in the concentration of 10^9^ colony forming units (CFU)/mL in Luria Bertani (LB) medium and *E. faecalis* in brain-heart infusion (BHI) medium was consecutively infused into each hole in the bone while taking care not to overfill the holes (Figure 1a). The beaker was covered with a Petri dish to keep the bone hydrated and held at room temperature in darkness for 24 h. Then, the presence of viable bacteria in all of the holes was verified by sampling with inoculating loop and subsequent microscopic observation. If the presence of bacteria in the holes was not proved, the bone sample was discarded. This happened in 3% of cases. After that, usually two holes were filled with the local carrier blended with the peptide (or with a mix of two peptides) and the remaining two holes were filled with the carrier alone and with antibiotic. The ChronOS Inject carrier was used as a two-component product supplied by the company Synthes GmbH, Oberdorf, Switzerland. The powder component consists of calcium phosphate blended with additives and the liquid component is a 0.5% sodium hyaluronate aqueous solution. The paste for filling the holes was prepared by successively mixing the powder component (200 mg) with the peptide (10 mg) or antibiotic (10 mg) and the liquid component (70 μL). Note that, in terms of molarities, the amount of antibiotic in the hole was higher than the amount of the peptide. The amount of resulting paste was sufficient to fill the entire space of the hole. The ratio between the powder component and the antimicrobial compound corresponds to the ratio used by other researchers for filling the infected bone cavity in rabbits [24]. The paste without peptides used as a control was prepared in the same manner (Figure 1b). After two days at room temperature, the fillings were thoroughly removed using a surgical spoon and the empty holes (Figure 1c) were thoroughly wiped out using sterile wet swabs. The swabs were extracted with physiological solution (400 μL), and the extracts were tenfold serially diluted and plated onto LB agar in Petri dishes (Figures S1–S3). Following overnight incubation, the numbers of bacterial colonies were counted. The bacterial load was expressed as CFU per hole. The infected femur heads used were deactivated by autoclaving and then disposed of as biological waste at the Department of Pathology, University Hospital in Motol.

### 4.5. AMPs Preventing Biofilm Formation on Surface of Model Implants

The bone samples with bored holes inside were prepared and handled as above. The bone cement (Palacos®r, Hereaus Medical GmbH, Wehrheim, Germany) with AMP incorporated was prepared by swift and thorough mixing of the polymer powder component (poly-methylacrylate, methyl methacrylate, 125 mg) and AMP (10 mg) or antibiotic (10 mg) with the liquid monomer (methyl methacrylate, *N*,*N*-dimethyl-p-toluidine, hydroquinone and colorant E141, 70 μL). The resulting elastic paste was pressed using a 2 mL plastic syringe into two bored holes, where it polymerized and solidified. The paste without peptide for filling of the remaining two holes was prepared similarly by mixing the powder component (125 mg) and liquid component (68 μL). In one of the two experimental setups, the cements were implanted inside the infected holes. In the second experimental setup, the bacteria were pipetted into a hole made between the cement implants (placed in the non-infected holes) from which they infiltrated towards the implants (Figure 3). After two days, the implants were taken out. Any bone debris sticking to them was manually removed. The implants were then washed four times with physiological solution to remove non-adhered bacteria. After that, the implants were sonicated in 0.5 mL of physiological solution in order to disrupt the bacterial biofilm deposited on their surfaces. The bacteria released from the biofilm into the solution were tenfold serially diluted and plated onto the agar in Petri dishes in order to count their colonies (Figure S4 as an example).

### 4.6. Statistical Analysis

The experimental data were obtained at least from three independent measurements and expressed as means ± standard error of mean (SEM). The statistical differences between results were calculated using one-way analysis of variance (ANOVA) using GraphPad Prism 5 software (GraphPad Software, Inc., La Jolla, CA, USA) and a *p*-value < 0.05 was considered significant.

## 5. Conclusions

This study shows that the controlled release of AMPs from carrier materials might be a promising alternative strategy for treating osteomyelitis and preventing implant-related infection in orthopaedics due to several potential advantages of AMPs. In addition, AMPs proved superior to current antibiotics against causative strains of osteomyelitis when examined in our model of induced osteomyelitis. Regrettably, this is an *ex vivo* study that does not completely reproduce the situation under the physiological conditions of living bone tissue. The experiments are time-consuming. The bone samples showed different qualities in terms of physical features and medical history, thus making statistical evaluation problematic. On the other hand, this model represents a new *ex vivo* laboratory assay that is more relevant for an efficacy evaluation of antimicrobials locally delivered from carriers into infected bone tissue than the standard in vitro testing of antimicrobial activity performed in test tubes. It is the first study that compares the effect of antimicrobial peptides with antibiotics when released from carriers or bone cement to infected bone samples.

## Figures and Tables

**Table 1:** Amino acid sequences, mass spectrometry data, and retention times of peptides.

Peptide	Sequence	Molecular Mass (Da)		t_R_ (min)
		Calculated	Found	
I	GKWMKLLKKILK-NH_2_	1483.98	1484.0	31.74
II	**GKWMKLLKKILK**-NH_2_	1483.98	1484.3	31.74
III	GKWKKLLKKILK-NH_2_	1481.03	1481.0	26.59
IV	GKWMKMLKKILK-NH_2_	1501.94	1501.9	30.23
V	GKWLKLLKKILK-NH_2_	1466.02	1466.7	32.84
VI	GKWVKLLKKILK-NH_2_	1452.01	1452.0	30.34
VII	**GKWVKLLKKILK**-NH_2_	1452.01	1452.0	30.34
VIII	GKWMKLLKKILK-NH_2_	1483.98	1484.0	27.59
IX	KWMKLLKKILK-NH_2_	1426.96	1427.0	27.82
X	**KWMKLLKKILK**-NH_2_	1426.96	1427.0	27.82
XI	βAKWMKLLKKILK-NH_2_	1497.99	1498.5	31.63
XII	β**AKWMKLLKKILK**-NH_2_	1497.99	1498.2	31.63

βA, beta-alanine; d-amino acids are shown in bold letters.

**Table 2:** Antimicrobial and haemolytic activity of peptides I–XII and antibiotics.

Antimicrobial Activity MIC (μmol/L)									Haemolytic Activity LC_50_ (μmol/L) ^c^
Peptide	MRSA 6271	MRSA ^a^	MRSA ^b^	*S. e.* ^b^	*E. f.* ^b^	*P. a.* 5482	*P. a.* ^a^	*C. a.*	
I	6.3	3.6	5.7	2.3	13.3	2.8	10.0	5.7	339
II	11.3	5.3	9.7	2.0	12.7	4.5	12.5	5.7	>400
III	29.7	11.5	25.3	4.7	>100	2.9	9.5	9.5	>400
IV	20.0	10.0	20.0	3.2	>100	5.3	12.7	12.7	>200
V	10.0	8.0	10.0	2.2	50.7	4.0	10.7	16.0	196
VI	9.0	5.0	10.0	2.5	>100	3.0	12.7	6.3	>400
VII	22.0	15.0	22.0	4.2	25.3	4.5	25.3	12.5	>400
VIII	18.7	13.3	32.0	4.0	>100	5.0	32.0	8.0	>400
IX	13.5	8.0	17.7	3.2	>100	2.9	5.8	9.5	>400
X	19.3	14.0	14.0	3.5	36	4.5	18.0	11.3	>400
XI	11.3	5.7	10.0	2.8	59.0	2.8	7.0	11.3	270
XII	16.0	8.0	16.0	2.5	32.0	3.2	8.0	5.3	>400
Vanc	0.9	1.1	1.1	1.5	1.3	>200	>320	-	-
Gent	325	3.9	4.5	109	95.2	2.0	>220	-	-

MRSA, methicillin-resistant *Staphylococcus aureus*; *S. e.*, *Staphylococcus epidermidis*; *E. f.*, *Enterococcus faecalis*; *P. a.*, *Pseudomonas aeruginosa*; *C. a.*, *Candida albicans*; ^a^ Clinical isolates from Liberec Regional Hospital; ^b^ Clinical isolates from University Hospital in Motol, Prague; ^c^ Concentration causing lysis of 50% of red blood cells. Vanc, vancomycin; Gent, gentamicin; each peptide was tested at least three times in duplicates.

**Table 3:** Effect of selected AMPs on eradication of infection inside the bone tissue.

Bacteria	Colony Forming Units (CFU)/Hole			
	ChronOS Alone	ChronOS with Peptide No	ChronOS with Antibiotic	
*MRSA 6271*	7.0 × 10^7^	(I) 7.6 × 10^2^	N/A	
*MRSA 6271*	4.0 × 10^5^ (3 × 10^7^)	(I + II) 2.0 × 10^3^	Vancomycin	2.0 × 10^5^
*MRSA 6271*	2.1 × 10^6^	(XII) 0	Vancomycin	1.3 × 10^6^
*MRSA* ^a^	1.2 × 10^6^	(I + II) 0	N/A	
*MRSA* ^a^	1.6 × 10^6^	(I + II) 4.0 × 10^2^	N/A	
*MRSA* ^a^	2.8 × 10^6^	(I + II) 0	N/A	
*MRSA* ^a^	1.2 × 10^7^ (6 × 10^8^)	(I + II) 4.8 × 10^3^	Vancomycin	6.0 × 10^6^
*MRSA* ^a^	2.0 × 10^6^	(IV) 2.5 × 10^3^	Ceftriaxone	4.1 × 10^6^
*MRSA* ^a^	3.4 × 10^7^	(I) 2.3 × 10^5^/(IX) 2.3 × 10^5^	N/A	
*MRSA* ^a^	3.9 × 10^6^	(VI) 0/(IX) 1.3 × 10^4^	Gentamicin	2.9 × 10^5^
*MRSA* ^a^	1.4 × 10^7^	(X) 1.8 × 10^4^	Vancomycin	1.4 × 10^7^
*MRSA* ^b^	7.0 × 10^7^	(I + II) 5.4 × 10^2^	Gentamicin	9.2 × 10^5^
*MRSA* ^b^	4.8 × 10^6^	(XI) 4.0	N/A	
*MRSA* ^b^	5.0 × 10^5^	(XI + XII) 0	N/A	
*S. e.*	1.2 × 10^5^	(I) 0/(III) 0	N/A	
*S. e.*	7.2 × 10^4^ (2.4 × 10^7^)	(V) 1.2 × 10^2^	Gentamicin	6.0 × 10^3^
*S. e.*	9.5 × 10^4^	(VI + VII) 0	N/A	
*P. a. 5482*	1.0 × 10^9^	(I + II) 4.0 × 10^4^/(XI) 2.0 × 10^5^	N/A	

Each row represents the results of a single experiment performed on one femoral head; ^a^ Clinical isolates from Liberec Regional Hospital; ^b^ Clinical isolates from University Hospital in Motol, Prague; (+) the hole was filled with ChronOS Inject calcium phosphate cement containing a mixture of two different peptides; /different holes in the same femoral head were filled with ChronOS containing different peptides; N/A, not applied. The CFU values shown in brackets in the second column correspond to the control hole, which was not filed with the paste. See Figures S1–S3.

**Table 4:** Effect of selected peptides incorporated into bone cement on the prevention of microbial adhesion and consequent biofilm formation on its surface.

Microbe	CFU/Implant			
	Palacos®r without Peptide	Palacos®r with Peptide No	Palacos®r with Antibiotic or Antimycotic	
*MRSA 6271*	6.0 × 10^4^	(VI) 0	N/A	
*MRSA 6271*	2.0 × 10^6^	(VI) 8.6 × 10^1^	N/A	
*MRSA 6271*	6.9 × 10^4^	(IX) 0/(IX) 0	Vancomycin	6.9 × 10^4^
*MRSA 6271	3.3 × 10^5^/1 × 10^6^	(VI) 1.4 × 10^2^/(VI) 0	N/A	
*MRSA* ^a^	7.0 × 10^6^	(IV) 0/(IV) 0	N/A	
*MRSA* ^a^	8.8 × 10^3^	(IX) 0/(IX) 0	N/A	
*MRSA* ^b^	7.5 × 10^6^/3.5 × 10^6^	(VI) 2.5 × 10^2^/(VI) 1.0 × 10^1^	N/A	
*MRSA ^b^	1.2 × 10^5^/0.6 × 10^5^	(I) 0/(I) 0	Vancomycin	4.0 × 10^6^
*S. e.*	6.0 × 10^4^/3.2 × 10^3^	(VI) 4.0 × 10^2^/(VI) 0	N/A	
*E. f.*	2.9 × 10^5^	(I) 0/(I) 0	N/A	
*E. f.	2.8 × 10^6^	(I) 0	Vancomycin	4.0 × 10^6^
*P. a.* 5482	1.6 × 10^6^/4 × 10^6^	(I) 5 × 10^1^/(I) 4	N/A	
*C. albicans* ^c^	4.4 × 10^5^	(VIII) 6.0 × 10^3^	Fluconazole	3.6 × 10^4^
*C. albicans* ^d^	6.0 × 10^3^	(I) 4.0/(I) 0/(IX) 3.4 × 10^2^	N/A	
*C. albicans* ^d^	5.4 × 10^4^/1 × 10^5^	(VIII) 0/(VIII) 0	Amphotericin B	0
* C. tropicalis	3.7 × 10^5^	(I) 0/(I) 4.0	Amphotericin B	1.3 × 10^6^
*C. glabrata*	1.3 × 10^6^	(VIII) 2.4 × 10^3^	Clotrimazole	1.2 × 10^6^

Each row represents the result of single experiment performed on one femoral head; ^a^ Clinical isolates from Liberec Regional Hospital; ^b^ Clinical isolates from University Hospital in Motol, Prague; ^c^ ATCC MYA-2876; ^d^ F7-39/IDE99; /Two implants in the same bone sample. * In this experimental setup, cements were implanted inside infected holes. In remaining cases, microbes were pipetted into the hole made between the cement implants (placed into non-infected holes) from which they infiltrated towards the implants (see experimental part); N/A, not applied.

## Supplementary Materials

The following are available online at http://www.mdpi.com/1424-8247/11/1/20/s1, Figure S1: Effect of peptide V on the eradication of infection inside the bone sample compared to gentamicin, Figure S2: Effect of the mixture of peptides I and II on the eradication of infection inside the bone sample compared to vancomycin, Figure S3: Effect of the mixture of peptides I and II on the eradication of infection inside the bone sample compared to vancomycin, Figure S4: Peptide I prevents *P. aeruginosa* biofilm formation on Palacos®r, Figure S5: Identification of human defensins in bone marrow tissue (**a**) RP-HPLC profile of bone marrow extract at 220 nm; (**b**) electrospray ionization mass spectrometry (ESI-MS) spectrum of the human α-defensins, HNP1, HNP2, and HNP3.
