# Supplementary material for: Antimicrobial Peptides for Topical Treatment of Osteomyelitis and Implant-Related Infections: Study in the Spongy Bone
Source: Pharmaceuticals (Basel). 2018 Feb 16;11(1):20. doi: 10.3390/ph11010020 (PMC5874716; doi:10.3390/ph11010020)
Supplement: Supplementary file 1 [file pharmaceuticals-11-00020-s001.pdf]

Supplementary Material: Antimicrobial Peptides for Topical Treatment of Osteomyelitis and Prevention of Implant-Related Infections: Study in the Spongy Bone

Figure S1. Effect of peptide V on the eradication of infection inside the bone sample compared to gentamicin

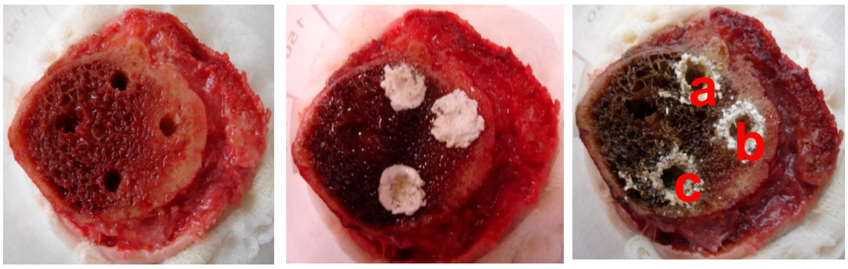

Staphylococcus epidermidis (clinical isolate from the University Hospital in Motol)

Peptide V vs. gentamicin loaded in ChronOS calcium phosphate cement

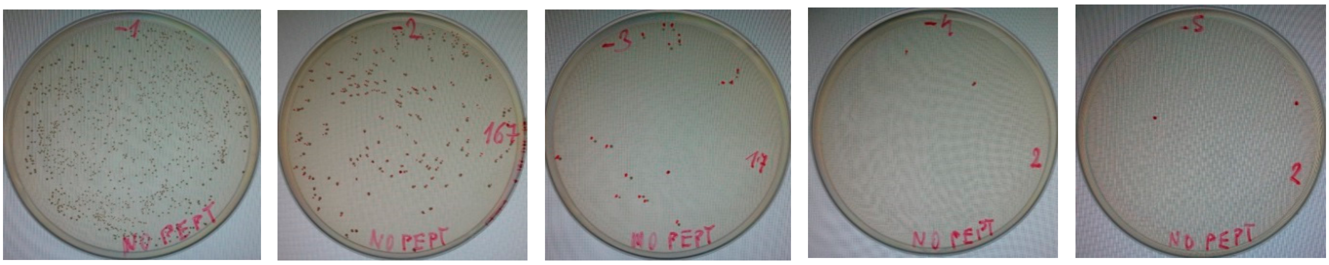

a) ChronOS without peptide

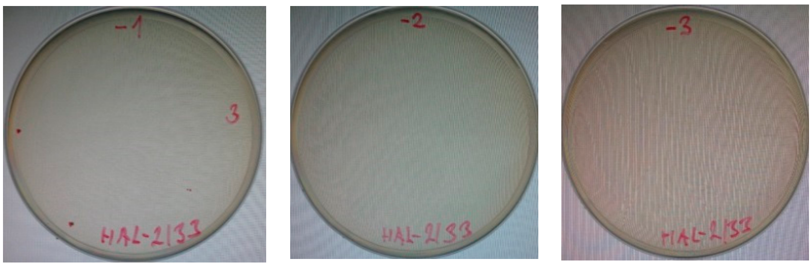

b) ChronOS with peptide

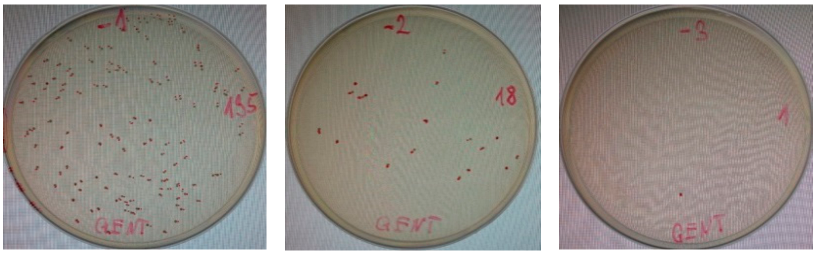

c) ChronOS with gentamicin

Figure S2. Effect of the mixture of peptides I and II on the eradication of infection inside the bone sample compared to vancomycin

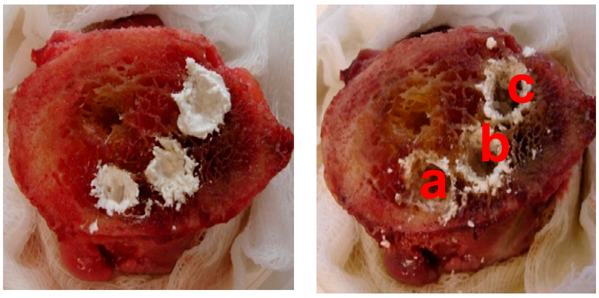

Staphylococcus aureus MRSA 6271

Peptide I + peptide II (4 : 1) vs. vancomycin loaded in ChronOS calcium phosphate cement

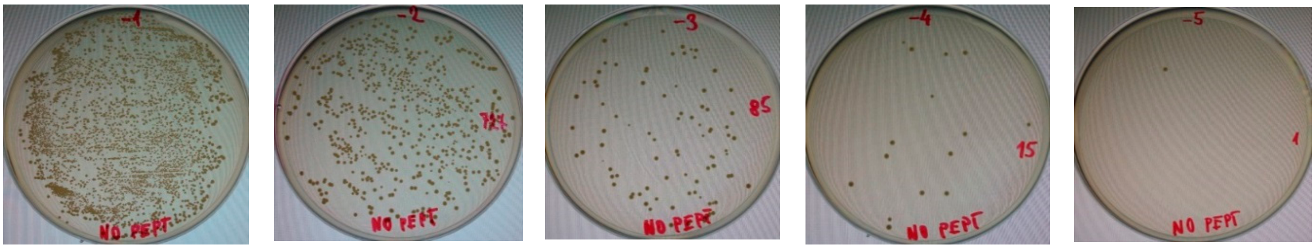

a) ChronOS without peptides

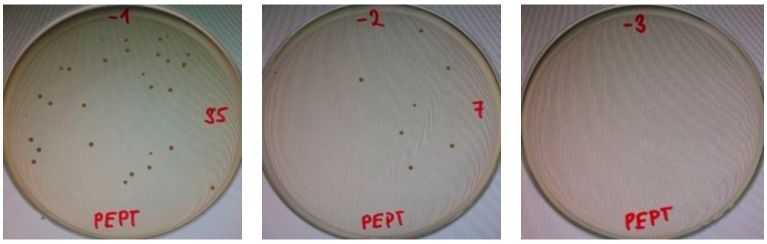

b) ChronOS with peptides

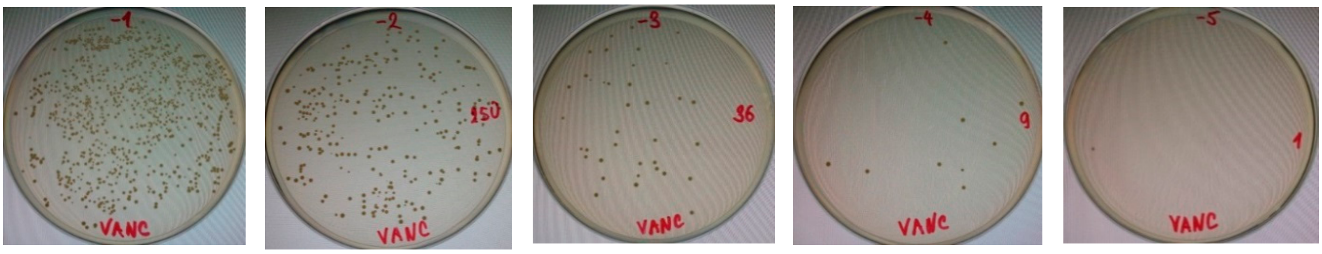

c) ChronOS with vancomycin

Figure S3. Effect of the mixture of peptides I and II on the eradication of infection inside the bone sample compared to vancomycin

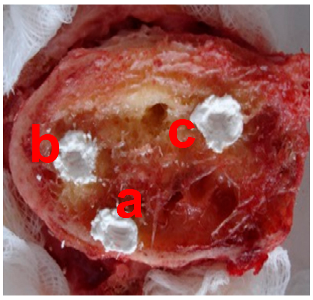

Staphylococcus aureus MRSA (clinical isolate from Regional Hospital in Liberec)

Peptide I + peptide II (4 : 1) vs. vancomycin loaded in ChronOS calcium phosphate cement

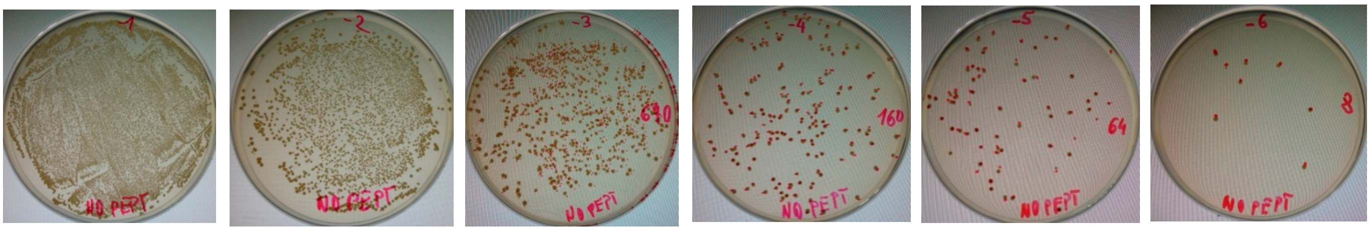

a) ChronOS without peptide

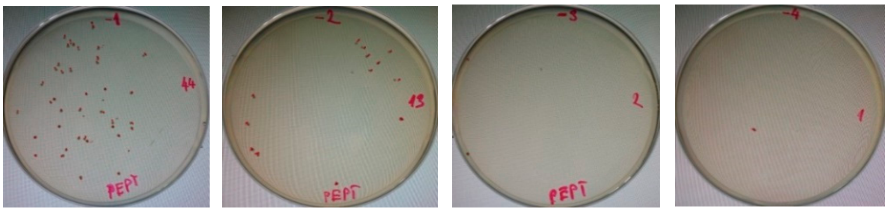

b) ChronOS with peptide

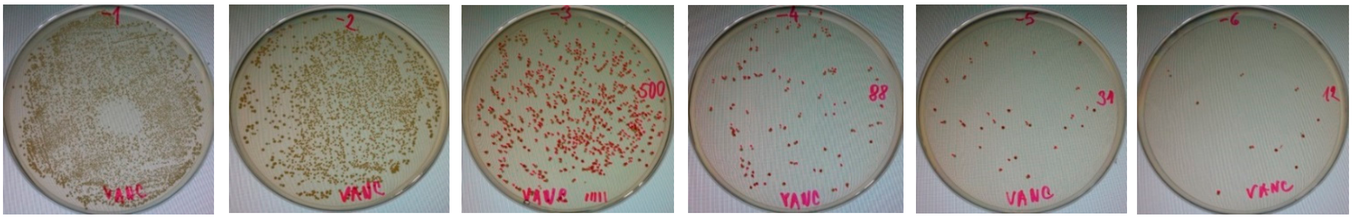

c) ChronOS with vancomycin

Figure S4. Peptide I prevents *P. aeruginosa* biofilm formation on Palacos®r

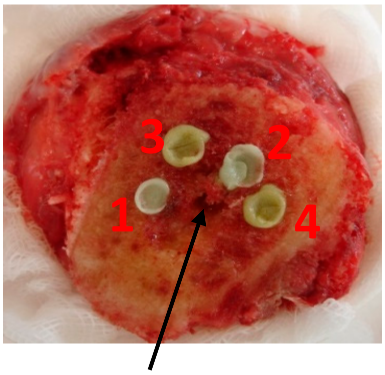

P. aeruginosa penetrated from the hole (arrow) towards implants made from Palacos®r

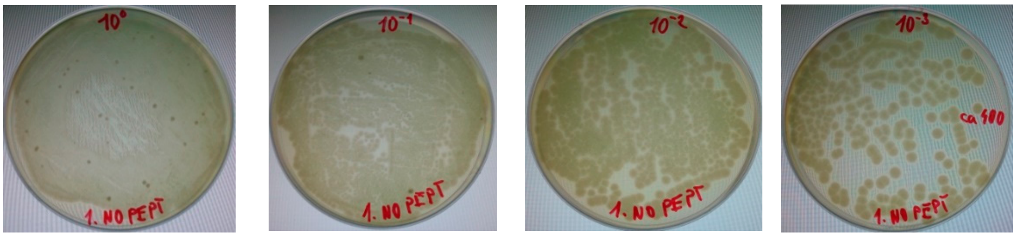

1. Palacos®r without peptide

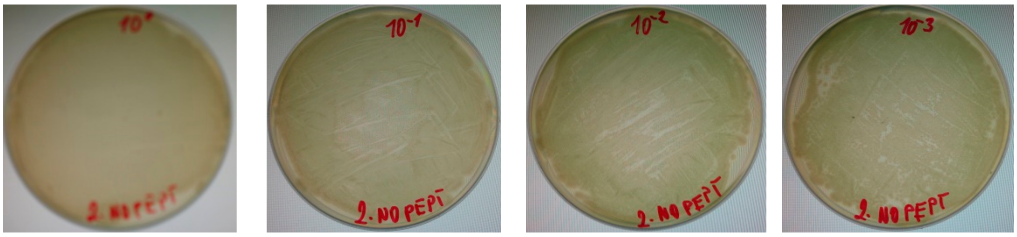

2. Palacos®r without peptide

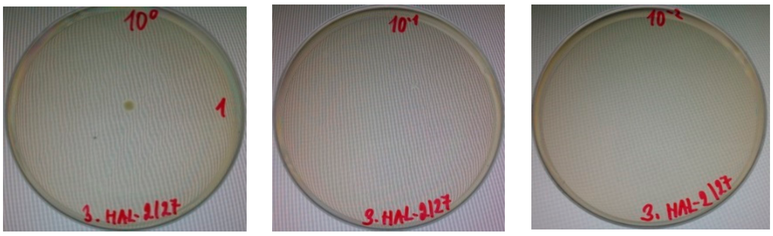

3. Palacos®r with peptide I

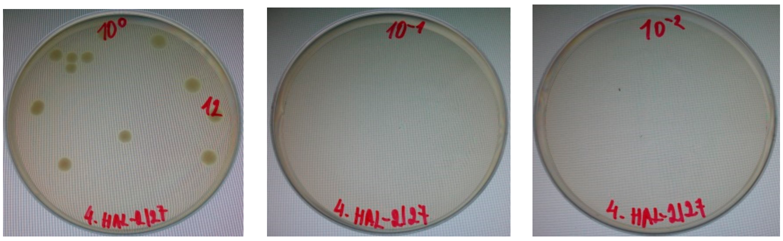

4. Palacos®r with peptide I

Figure S5. Identification of human defensins in bone marrow tissue

The sample of bone marrow tissue taken from femur head was extracted with acetonitrile-water (1:1) mixture containing 1% trifluoroacetic acid (TFA) and then ultrafiltered using Amicon centrifugal filter units 0.5 mL with a 50-kDa molecular weight cut-off membrane. The filtrate was lyophilized and then fractioned by RP-HPLC. The material of the peak eluted at 25.4 min was analyzed by mass spectrometry (Fig. S5b).

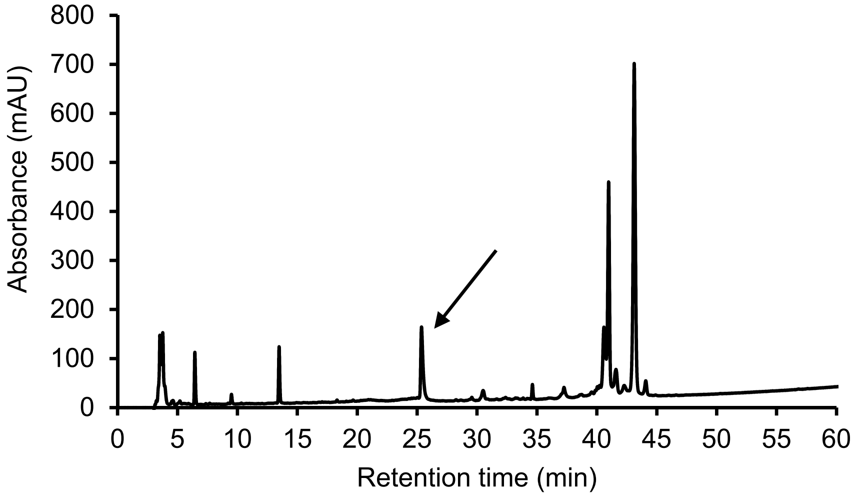

a) RP-HPLC profile of bone marrow extract at 220 nm.
An elution gradient of solvents from 5% to 70% acetonitrile-water-0.1% TFA was applied for 60 min at a flow rate 1 mL/min. Arrow indicates the peak containing the mixture of three human defensins (HNP1, HNP2, and HNP3).

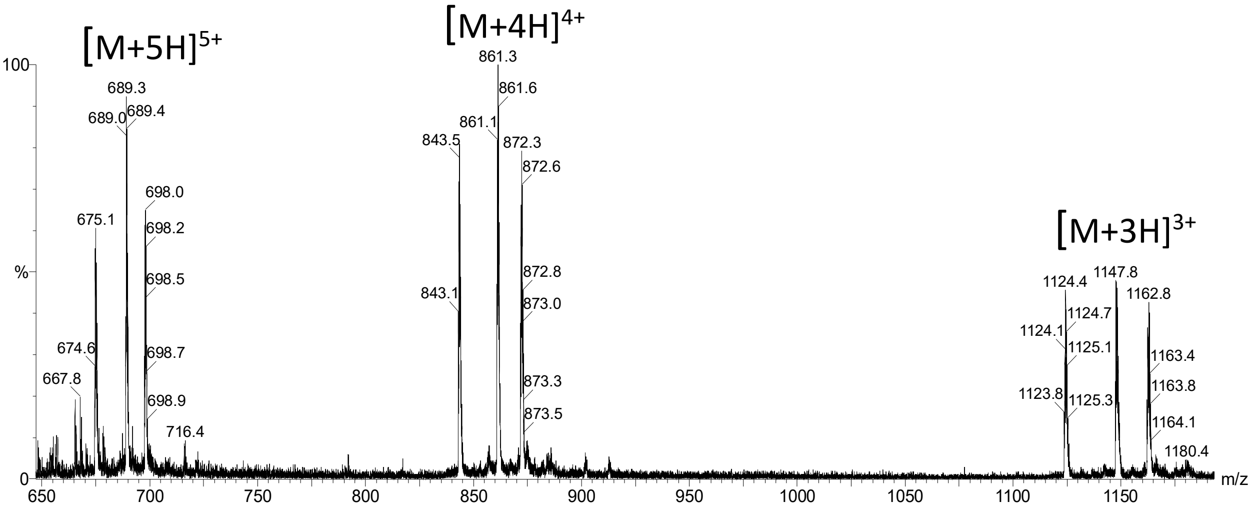

b) ESI-MS spectrum of the compounds corresponding to the peak of t_R 25.4 min (Fig S-5)

Mr = 3368.4 CYCRIPACIAGERRYGTCIYQGRLWAFCC (HNP2)
Mr = 3439.5 ACYCRIPACIAGERRYGTCIYQGRLWAFCC (HNP1)
Mr = 3483.1 DCYCRIPACIAGERRYGTCIYQGRLWAFCC (HNP3)
